# Anti-Hepatitis C Virus T-Cell Immunity in the Context of Multiple Exposures to the Virus

**DOI:** 10.1371/journal.pone.0130420

**Published:** 2015-06-24

**Authors:** Katja Pfafferott, Pooja Deshpande, Elizabeth McKinnon, Shahzma Merani, Andrew Lucas, David Heckerman, Simon Mallal, Mina John, Silvana Gaudieri, Michaela Lucas

**Affiliations:** 1 Institute for Immunology and Infectious Diseases, Murdoch University, Murdoch, Western Australia, Australia; 2 School of Anatomy, Physiology and Human Biology, University of Western Australia, Crawley, Western Australia, Australia; 3 Centre for Forensic Science, University of Western Australia, Crawley, Western Australia, Australia; 4 Microsoft Research, Microsoft, Redmond, Washington, United States of America; 5 Division of Infectious Diseases, Department of Medicine and Department of Pathology, Microbiology and Immunology, Vanderbilt University, Nashville, Tennessee, United States of America; 6 School of Medicine and Pharmacology, Harry Perkins Institute, University of Western Australia, Crawley, Western Australia, Australia; 7 School of Pathology and Laboratory Medicine, University of Western Australia, Crawley, Western Australia, Australia; KAIST, Graduate School of Medical Science & Engineering, REPUBLIC OF KOREA

## Abstract

Characterisation of Hepatitis C virus (HCV)-specific CD8^+^ T-cell responses in the context of multiple HCV exposures is critical to identify broadly protective immune responses necessary for an effective HCV vaccine against the different HCV genotypes. However, host and viral genetic diversity complicates vaccine development. To compensate for the observed variation in circulating autologous viruses and host molecules that restrict antigen presentation (human leucocyte antigens; HLA), this study used a reverse genomics approach that identified sites of viral adaptation to HLA-restricted T-cell immune pressure to predict genotype-specific HCV CD8^+^ T-cell targets. Peptides representing these putative HCV CD8^+^ T-cell targets, and their adapted form, were used in individualised IFN-γ ELISpot assays to screen for HCV-specific T-cell responses in 133 HCV-seropositive subjects with high-risk of multiple HCV exposures. The data obtained from this study i) confirmed that genetic studies of viral evolution is an effective approach to detect novel in vivo HCV T-cell targets, ii) showed that HCV-specific T-cell epitopes can be recognised in their adapted form and would not have been detected using wild-type peptides and iii) showed that HCV-specific T-cell (but not antibody) responses against alternate genotypes in chronic HCV-infected subjects are readily found, implying clearance of previous alternate genotype infection. In summary, HCV adaptation to HLA Class I-restricted T-cell responses plays a central role in anti-HCV immunity and multiple HCV genotype exposure is highly prevalent in at-risk exposure populations, which are important considerations for future vaccine design.

## Introduction

Hepatitis C virus (HCV) infection remains a major health problem worldwide. Although the recent development of direct-acting anti-viral (DAA) drugs has revolutionised the efficacy of treatment for hepatitis C, these new drugs will not prevent re-infection, which is a common occurrence in high-risk HCV exposure populations [1]. Accordingly, there is a continuing need for the development of a protective vaccine against circulating genetically diverse HCV genotypes (GTs).

The ability to develop a T-cell based vaccine against HCV should be bolstered by knowledge of the effective CD4^+^ and CD8^+^ T-cell responses that mediate natural immunity in humans [2]. However, the diversity of HCV strains and of host molecules that restrict antigen presentation (human leucocyte antigens; HLA) complicates our ability to understand host-viral interplay and has hampered progress in the development of a HCV vaccine.

Host HLA genes have been subject to positive selection from repeated exposure to infectious pathogens in our history and as such exhibit an extraordinary level of diversity at the population level that results in often non-overlapping sets of viral peptides presented by different individuals. Such diversity in antigen presentation within host populations makes it difficult to identify and assess HCV T-cell targets. The natural variation observed for HCV strains due to a high mutation rate leading to immune escape (adaptation) as well as repeat exposure to variant strains due to high risk behaviour adds an additional layer of complexity in host-viral interplay [3–5]. Although previous studies have identified a number of HCV T-cell targets, utilising peptides derived from reference strains, the breadth of HLA alleles and viral sequences examined in these studies tend to be narrow relative to the diversity of the HLA genes and circulating HCV strains within populations ([6], www.iedb.org). Furthermore, when using overlapping peptides in cellular assays the specificity of the HLA-restriction of the T-cell response is sometimes unclear due to the extensive HLA repertoire of subjects. As shown for HIV [7], HLA and viral diversity within a host population need to be considered when developing a T-cell based vaccine, however this analysis is lacking for HCV.

We previously performed a large population-based genetic study to identify allele specific HLA Class I-associated viral polymorphisms within the non-structural proteins of HCV in the context of GT1 and GT3 infection [8, 9]. These HLA Class I-associated viral polymorphisms represent amino acids selected by HLA Class I-restricted T-cell pressure and therefore are likely to mark true in vivo CD8^+^ T-cell targets or epitopes. As the genetic study identifies viral adaptations within circulating viruses in the population, it overcomes the limitation of previous cellular studies that commonly utilise peptides that are based on a reference or consensus sequence, which typically differ from diverse circulating viral strains. Accordingly, the genetic study enables the design of T-cell targets for cellular testing that allow comparison between adapted and non-adapted variants for a T-cell epitope. Furthermore, given the limited overlap in viral adaptation sites between GT1 and GT3 [9], a number of these T-cell targets are likely to represent HCV GT-specific T-cell epitopes.

There is also limited evaluation of the immune hierarchy of T-cell responses during HCV infection. Data on anti-HIV immunity has shown that HLA-B-restricted responses play a major role in the overall HIV-specific CD8^+^ T-cell response [10] and have the strongest effect on HIV infection outcome [11]. Furthermore, recent evidence suggests differences in the likely contribution of host HLA Class I-restricted responses between DNA and RNA viruses and also between the RNA virus genera flaviviruses (that contains HCV) and other RNA virus genera [11, 12]. However, data in regards to the contribution of HLA-A, -B and -C-restricted responses to the overall anti-HCV response during infection are missing, mainly due to the focus on a small set of immunodominant HLA-B-restricted epitopes and HLA-A*02-restricted T-cell epitopes in the literature.

Population-based genetic studies of viral adaptation provide leads on putative T-cell targets for all HLA class I loci without bias [8, 9, 13, 14]. In this study, predicted HLA Class I-restricted T-cell epitopes based on HLA Class I-associated viral polymorphisms were initially evaluated for their capacity to elicit ex vivo CD8^+^ T-cell responses. These peptides were then used to compare the contribution of HLA-A, -B and -C-restricted responses to the overall HCV-specific CD8^+^ T-cell response and to assess the extent of historic exposure to different GTs. Overall, this study presents data on anti-HCV T-cell responses, which accounts for circulating viral variation, GT specificity and HLA diversity in subjects with resolved and chronic infection after multiple HCV exposures.

## Materials and Methods

### Study Subjects

HCV-exposed individuals (n = 133; 10 spontaneous resolvers, 25 resolvers following pegylated IFN-α/ribavirin treatment, 93 subjects with chronic HCV infection and five subjects with unknown infection status—i.e. unknown treatment resolver or spontaneous resolver due to lack of clinical information) were recruited from tertiary hospitals in Western Australia between 2006–2012. Each subject was followed for up to three years with a maximum of four blood samples taken per year (Table 1). The HCV GTs within the cohort were GT1 (51.1%) and GT3 (29.3%) and 19.5% with unknown GT exposure. Fifty per cent of subjects were exposed to HCV via contaminated blood clotting factors (male subjects with X-linked Haemophilia) whereas the remaining 50% represented individuals that acquired HCV infection predominantly via intravenous drug use (IDU). For subjects with Haemophilia modelling shows the likelihood of infection with more than one viral strain [15] and data from studies on intravenous drug users (IVDU) show that the level of repeat exposure to diverse viral strains is high [16].

**Table 1:** Subject demographics and clinical data.

	n	%
**Outcome**		
Spontaneous resolver	10	7.5
Treatment resolver	25	18.8
Chronic	93	69.9
unknown *	5	3.8
**Genotype**		
1 ^^^	68	51.1
2 ^B^	2	1.5
3 ^^^	39	29.3
4 ^B^	1	0.8
unknown *	24	19.5
**Transmission mode**		
Blood product	67	50.4
Other^#^	66	49.6
**Gender**		
Female	34	25.6
Male	99	74.4

* information unknown due to limited clinical history or lack of viraemic plasma sample

^^^ includes a single individual co-infected with GT1 and GT3 strains

^#^ predominately IDU and Tattoo

### Ethics statement

Written, informed consent from all subjects was obtained for this study. Ethics approval for the conduct of this research was obtained from the Royal Perth Hospital Ethics Committee (EC2004/005). The protocol and the procedures of the study were conducted in conformity with the ethical guidelines of the World Medical Association Declaration of Helsinki.

### Viral HCV RNA extraction

Viral RNA was extracted from plasma using the COBAS AMPLICOR HCV Specimen Preparation Kit v2.0 (Roche) according to the manufacturer’s instructions.

### PBMC separation and DNA extraction

Peripheral Blood Mononuclear Cells (PBMCs) and DNA were obtained from whole blood. PBMCs were isolated using the Accuspin System-Histopaque method (Sigma) and DNA was extracted using the QIAmp DNA Blood Mini Kit (QIAGEN) according to the manufacturer’s guidelines.

### HLA Genotyping

Sequence-based four-digit HLA Class I typing was performed by direct DNA sequencing methods as previously described [8].

### HCV Genotyping

HCV GTs/subtypes were assigned by clinical tests using commercial assays (INNO-LiPA HCV II; Innogenetics) and confirmed by phylogenetic analysis as previously described [9].

### HCV sequencing

Sequencing of the HCV non-structural genes was performed as previously described [8, 9]. Briefly, RT-PCRs were performed using extracted viral RNA to amplify the non-structural regions of HCV. First-round products were used as templates in nested second-round PCRs using generic or GT-specific primers. Amplicons were sequenced using the BigDye Terminator v3.1 cycle sequencing kit (Applied Biosystems) according to manufacturer’s recommendations and electropherograms edited using Assign (Conexio Genomics). Mixtures were identified where the secondary peak was ≥20% of the major peak.

### Prediction of HCV peptides

Web-based HLA binding programs SYFPHEITI ([17]; www.syfpeithi.de) and BIMAS ([18]; www-bimas.cit.nih.gov/molbio/hla_bind) were used to predict HCV-specific HLA Class I-restricted T-cell epitopes based on a list of HLA-associated HCV polymorphisms (p<0.05) identified by our previous analysis of NS2-NS5B HCV sequences from chronic HCV GT1-infected individuals [8, 9]. Specifically, at least 10 amino acids either side of the site of HLA-association was used in the web-based HLA-binding prediction programs. In addition, T-cell epitopes were predicted using a statistical HLA-binding prediction model described in [19]. A total of 76 putative GT1 CD8^+^ T-cell targets were identified in this study. A selection of published HLA Class I-restricted HCV GT1 T-cell epitopes was also included in the IFN-γ ELISpot assays. In order to capture immune responses towards the circulating viral strains within the cohort, consensus and up to two variant versions of peptides were synthesised (Mimotopes) based on sequence data from the genetic study [9]. The set of peptides corresponding to the GT1 CD8^+^ T-cell targets and used in the IFN-γ ELISpot assays are listed in S1 Table. As there was little overlap in the HLA-associated HCV polymorphisms found in GT1 and GT3 [9], likely reflecting limited overlap in T-cell pressure on the HCV genome (or sites of viral adaptation), the CD8^+^ T-cell epitopes identified in this study are likely to be GT-specific. However, peptides that elicit cross GT-reactive responses cannot be excluded.

Binding score cut-offs for the two web-based programs were determined relative to the median score for the known HCV CD8^+^ T-cell epitopes in S1 Table. Accordingly, a good binder was based on a prediction score above 20 for SYFPHEITI and/or 100 for BIMAS. Retrospectively, approximately a third of the putative epitopes were predicted to be good or intermediate binders by the IEDB HLA binding prediction program (<500 IC_50_; [20]; http://tools.immuneepitope.org/mhci/). However, more than half (57.8%) of the peptides deemed to be a non-binder with IEDB elicited a response in the IFN-γ ELISpot (based on criteria described below). As these HLA-associated viral polymorphisms would only be evaluated using IFN-γ ELISpot assays, predictions of processing mutations is not shown.

### IFN-γ ELISpot assays

ELISpot assays were performed using the Biomek FX liquid-handling system (Beckman Coulter) as previously described [21]. The protocol was adjusted to optimise the detection of HCV-specific T-cell responses as follows: PBMCs were added to an IFN-γ (Mabtech) pre-coated 96 well ELISpot plate (MAIPS, Millipore) at a concentration of 200,000 cells/well and incubated overnight with HCV peptides (final concentration of 10μl/ml). At least one well per study subject was allocated as a positive control (anti-CD3 antibody, Mabtech) and three wells as negative (autologous PBMC cells only) controls.

### Individualised HLA-based analysis of HCV-specific T-cell responses

We deemed a predicted HLA Class I-restricted T-cell epitope a true in vivo target if any of the corresponding peptides (consensus or variant) elicited an IFN-γ T-cell response at ≥25 spot forming units (SFU)/million PBMCs after background subtraction [22]. The background was defined as the mean plus three times the standard deviation of the number of spots counted in the triplicate negative control wells. The median background was 7.0. Given the large number of peptides tested for each individual, peptides were tested as singletons. However, of the 53 epitopes that were deemed positive using this approach (S1 Table), 18 were known HCV CD8^+^ T-cell epitopes and of the new targets identified six were positive in at least two independent IFN-γ ELISpot assays for the same subject and a further 14 were positive in at least two subjects. Given the large number of peptides tested for each subject, confirmatory ELISpot assays or intracellular cytokine staining using flow cytometry was not possible.

Individuals sharing a particular HLA allele were tested with the same set of HLA-matched peptides and peptides had to be tested in a minimum of five HLA-matched individuals to be included in the final analysis. Each epitope-specific response rate is calculated as the proportion of the tested individuals who yielded a positive response to that epitope (≥25 SFU/million PBMCs).

The response rate of CD8^+^ T-cell epitopes normalises for the bias that could be introduced in the data set by i) larger sample sizes of study subjects carrying common HLA alleles, and ii) variation in the number of peptide variants spanning the same T-cell epitope. Assessment of relative CD8^+^ T-cell epitope response rates across proteins and the HLA loci was undertaken by nested mixed effect modelling of individual responses within a generalized linear regression framework to accommodate the within-individual correlations.

### Analysis of antibody responses to different GTs

Serum samples from 73 subjects were tested with a commercial “serotyping” assay (Murex Biotech Limited) in order to find evidence for antibody responses to more than one GT. Briefly, immunoplates were precoated with synthetic peptides derived from the NS4 protein from HCV GT1-6. Diluted serum samples were added in the presence of competing peptides to block cross-reactivity. Captured antibodies were then detected using an anti-human IgG antibody enzyme complex with resulting colour read at 450nm absorbance and results analysed following the manufacturer’s instructions.

## Results

### Reverse genomics approach identifies novel HCV T-cell targets

Using leads from a population-based genetic study [8, 9] in combination with web-based binding programs (see Methods for details), we predicted 76 HLA Class I-restricted HCV GT1 T-cell epitopes of which 16 had previously been described (S1 Table). Peptides corresponding to these putative T-cell epitopes and an additional 16 previously published HLA Class I-restricted HCV GT1 T-cell epitopes were tested using IFN-γ ELISpot assays. The panel of peptides used in each assay was customised to the HLA repertoire of each individual in the cohort (n = 133), which consisted of subjects with chronic HCV infection and spontaneous or treatment-induced resolution (Table 1). The median SFU/million PBMCs was 63 with an interquartile range of 38.5–117.

This approach confirmed a total of 35 previously unknown HLA Class I-restricted GT1 T-cell epitopes comprising 14 HLA-A-, 17 HLA-B- and 4 HLA-C-restricted T-cell epitopes (S1 Table). Of these, four GT1 T-cell epitopes included a viral adaptation that was present in the most common circulating (consensus) viral sequence (indicated by an odds ratio of <1 in the genetic analysis; [8, 9]). Accordingly, peptides with the consensus sequence at these sites would effectively equate to testing for a T-cell response against escaped variants and epitopes may have been missed if a consensus-based approach had been used to identify T-cell epitopes.

Overall, the proportion of predicted HCV T-cell epitopes that elicited a response was similar to that observed for known HCV T-cell epitopes. Specifically, 19/23 (82.6%) predicted HLA-A epitopes versus 10/15 (66.7%) known HLA-A epitopes (of which six targets were the same between predicted and known) elicited an IFN-γ response in at least one subject while 23/44 (52.3%) predicted HLA-B epitopes versus 6/11 (54.5%) known HLA-B epitopes (of which 10 targets were the same between predicted and known) elicited an IFN-γ response in at least one subject. Four out of nine predicted epitopes for HLA-C elicited a response in at least one subject while none of the two known HLA-C epitopes elicited a response in any of the tested subjects. These results confirm that the reverse genomics approach described here is an effective tool to identify true in vivo T-cell targets.

### HLA-A-restricted immune responses are prevalent in the overall immune response against HCV GT1

**Fig 1:**
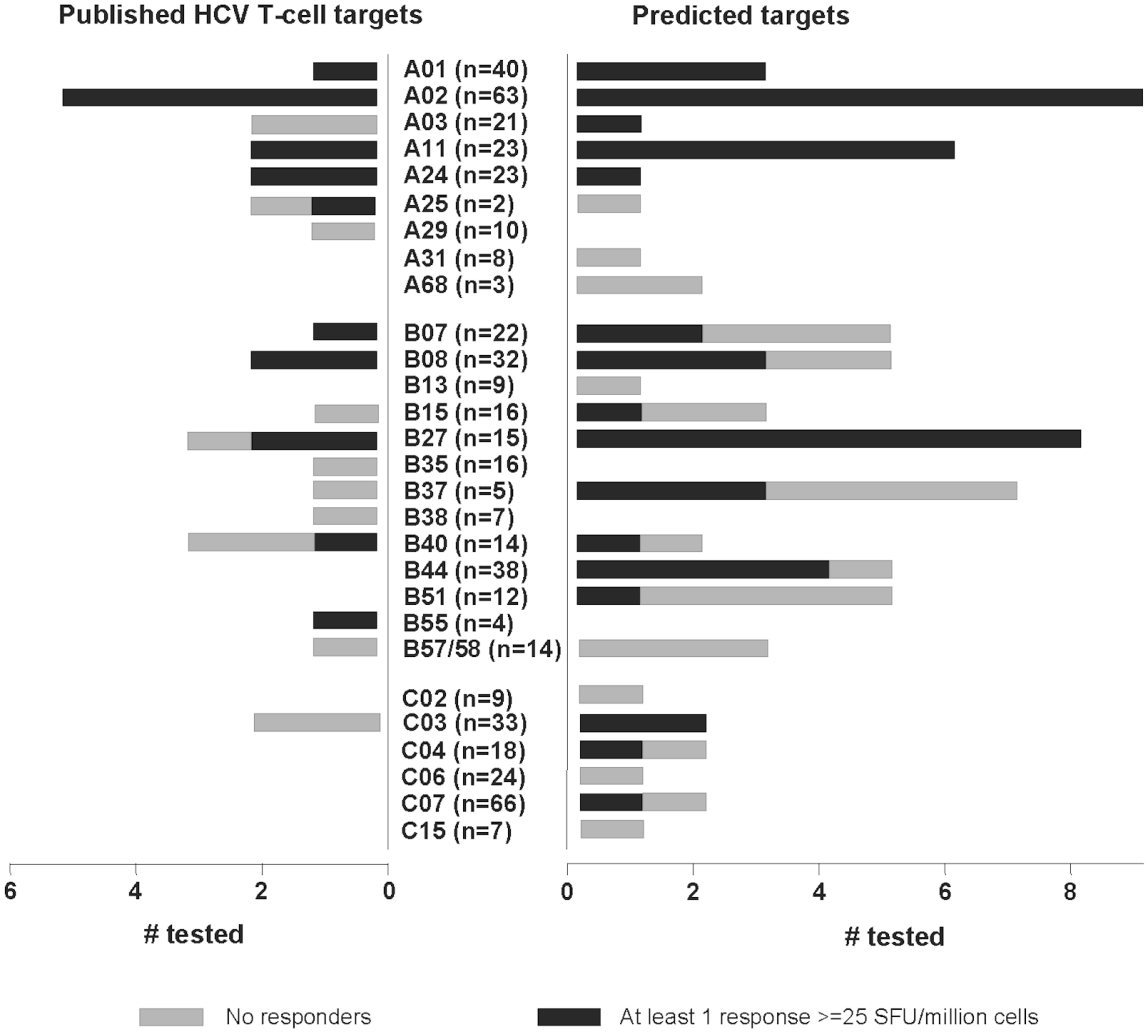
Breakdown of HLA-specific T-cell targets tested and number eliciting a T-cell response based on an IFN-γ ELISpot assay. Predicted T-cell targets include published HCV T-cell targets that contain a site associated with a specific HLA allele with the same restriction. Number in bracket indicates number of subjects tested that carry the particular HLA type.

In previous population-based genetic studies of HCV, we identified more HLA-B than HLA-A associated HCV polymorphisms [8, 9], which is similar to studies examining these statistical associations from single source HCV outbreaks [23, 24]. Based on the predictions from this genetic study, a greater number of HCV T-cell targets restricted by HLA-B (n = 44) were tested relative to HLA-A (n = 23). Nevertheless, the majority of HLA-A-restricted T-cell epitopes generated an immune response in the cohort tested, but many of the predicted HLA-B-restricted T-cell epitopes did not elicit IFN-γ responses (eight HLA-A restricted versus 29 HLA-B-restricted targets did not elicit a response in at least one individual at ≥25 SFU/million PBMCs; Fig 1).

**Fig 2:**
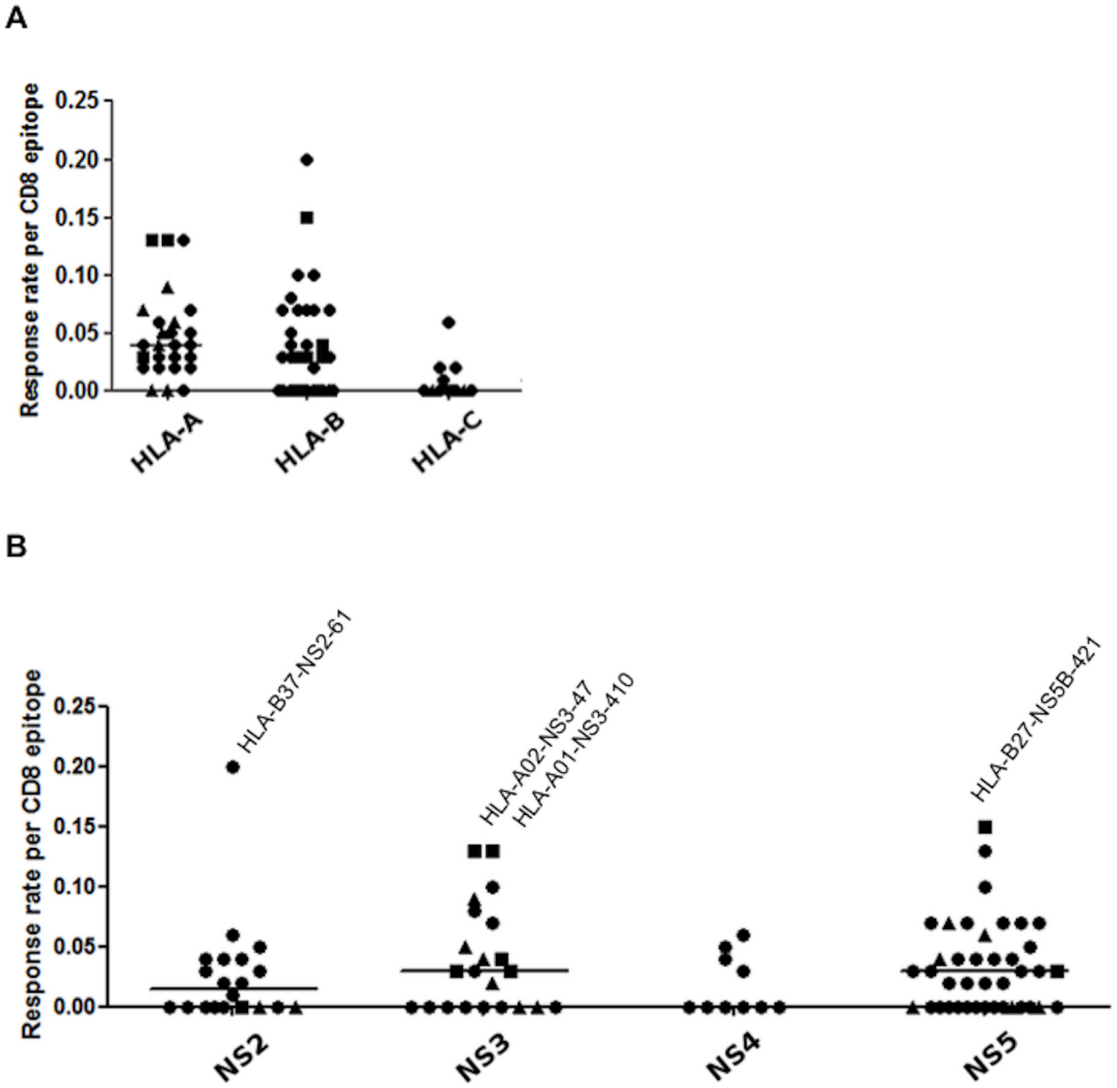
A. Contribution of HLA-A-, -B- and -C-restricted responses to overall anti-HCV immunity in the cohort. Response rates of predicted (circle), published with adaptation (square) and published (triangle) HCV CD8^+^ T-cell epitopes are shown in relation to the HLA loci. HLA-A-restricted T-cell epitopes have a significantly higher response rate compared to HLA-B- and -C-restricted T-cell epitopes (p = 0.008 and p = 0.0007, respectively). B. Response rate of T-cell epitopes within the HCV non-structural proteins. Predicted (circle) T-cell epitope, published T-cell epitope with adaptation (square) and published (triangle) T-cell epitopes that elicit an IFN-γ response. T-cell epitopes with the highest response rates are indicated. There was a greater response rate in NS3 relative to the other proteins (p = 0.02).

To take into account the discrepancy in subject numbers for the different HLA alleles, the efficiency of peptides in triggering an IFN-γ CD8^+^ T-cell response was evaluated based on a response rate per CD8^+^ T-cell epitope (see Methods). HLA-B- and -C-restricted epitopes were less likely to elicit a response than HLA-A-restricted epitopes (OR 1.63 (1.14–2.35, 95% CI) p = 0.008 and 4.28 (1.84–9.95) p = 0.0007, respectively, Fig 2A). As well, HLA-B-restricted T-cell epitopes were more likely to elicit a response than HLA-C-restricted T-cell epitopes (OR 2.62 (1.10–6.25, 95% CI) p = 0.03). It should be noted that the overall number of predicted T-cell epitopes obtained for the HLA-C alleles using the approach here remains small, partly due to the limited information known about the binding capabilities of different HLA-C alleles.

### Identification of commonly targeted HCV GT1 CD8^+^ T-cell epitopes in the non-structural proteins

The highest response rates were predominately seen towards previously described T-cell epitopes HLA-A*02-NS3-1072 (CINGVCWTV), HLA-A*01-NS3-1436 (ATDALMTGY), HLA-B*27-NS5B-2841 (ARMILLTHF) and HLA-B*37-NS2-870 (RDAVILLM) (Fig 2B) that all showed evidence of adaptation based on the genetic analysis. Interestingly, some of these T-cell epitopes, such as HLA-A*01-NS3-1436 (ATDALMTGY; [25]) and HLA-B*27-NS5B-2841 (ARMILLTHF; [4]) have been previously termed “immunodominant”.

When comparing the response rates of the HLA Class I-restricted T-cell epitopes tested across the different viral proteins, T-cell epitopes within the more conserved NS3 and NS5 did tend to have a higher response rate than those within NS2 and NS4 (Fig 2B). This trend only reached statistical significance in NS3 (OR 1.55 (1.07–2.24, 95% CI) p = 0.02). Of note, many of the T-cell epitopes that elicited a response in NS3 and NS5B have previously been described while many of those within the other non-structural HCV proteins are novel (S1 Table).

### Immune responses against adapted virus common in chronic HCV infection

Of the 29 T-cell epitopes that were tested with both the adapted and non-adapted form and elicited a response in chronic HCV GT1-infected subjects, 11 T-cell epitopes were only recognised when presented in their adapted form and would not have been detected using wild-type peptides. Of the remaining 18 T-cell epitopes, 12 were recognised in both the non-adapted and adapted form. For these 12 T-cell epitopes, 15 of 17 chronic HCV GT1-infected subjects had higher IFN-γ responses to the adapted form compared to the non-adapted form and of these subjects four had more than a two-fold difference in responses but there was no significant difference overall between responses to adapted and non-adapted peptides (Mann-Whitney, p = 0.2; median difference 42 SFU/10^6^ PBMCs). Six T-cell epitopes were only recognised in the non-adapted form.

### Individuals at high-risk of multiple HCV exposure are able to mount CD8^+^ T-cell responses against previously encountered HCV GT strains

**Fig 3:**
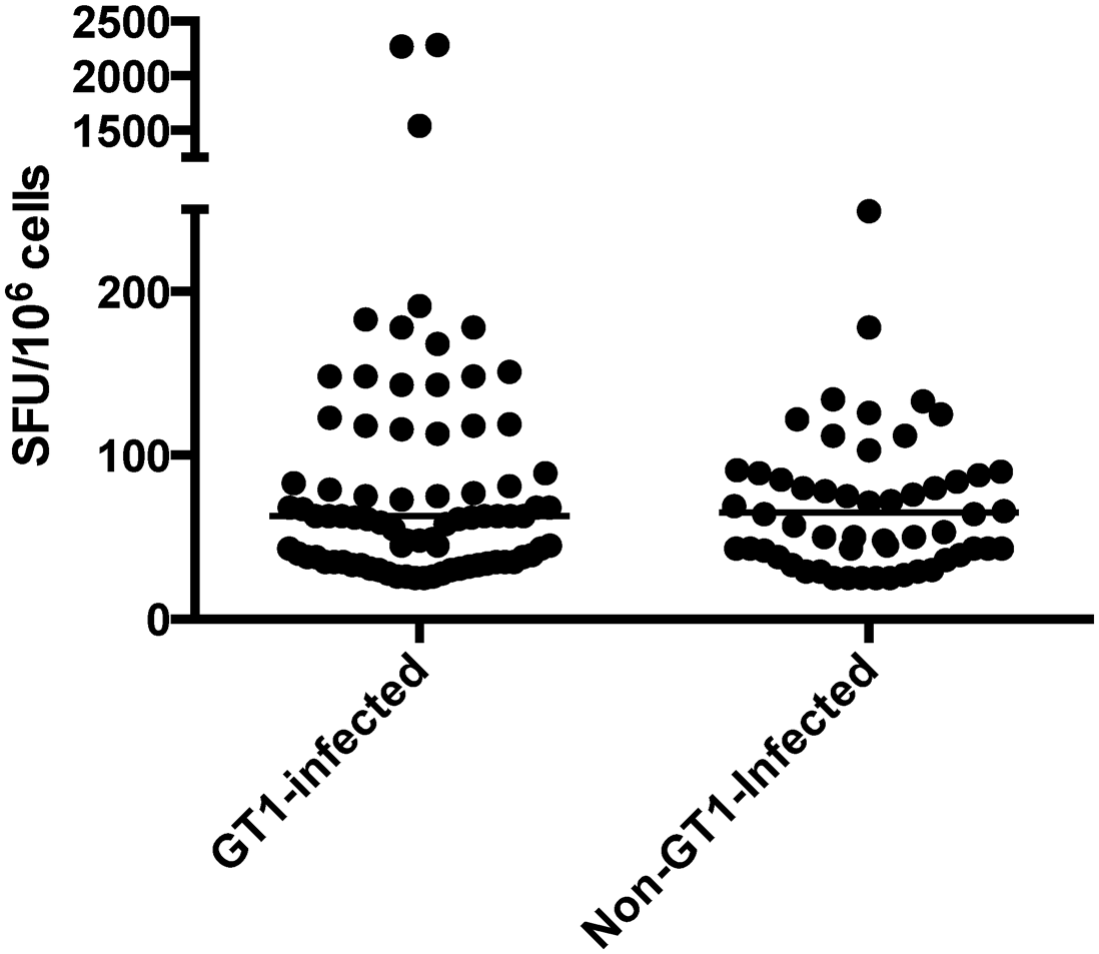
Chronic non-GT1-infected individuals are able to mount T-cell responses to GT1 epitopes. The SFU/million PBMCs are shown for subjects with known GT1 infection and non-GT1 infection. Although subjects may have responded to more than one peptide covering an epitope, in this figure only the highest response was used.

Most subjects in this study have been repetitively exposed to the different circulating HCV GTs due to the nature of their exposure. Therefore, chronic HCV-infected subjects currently infected with non-GT1 strains were screened for T-cell responses against GT1 peptides to reveal T-cell responses mounted during potentially previously resolved GT1 infection (Fig 3). Using this approach, 37.9% (11/29) of chronic non-GT1-infected subjects responded to peptides in the HCV GT1 peptide panel. This finding suggests that GT1 RNA-negative subjects may have previously successfully cleared GT1 virus and maintained a memory T-cell response against the resolved GT1 infection.

Interestingly, of the 35 novel HCV GT1 T-cell epitopes identified in this study peptides corresponding to 13 epitopes elicited a response only from non-GT1-infected subjects; six were restricted by HLA-B27—a HLA allele associated with good outcome following HCV infection [4, 26, 27] (Table 2). For most of the GT1 epitopes that elicited a response in non-GT1 infected subjects, the corresponding GT3 sequence reduced predicted binding scores, suggesting these are likely to be GT1-specific responses. Furthermore, some of the responses in non-GT1-infected subjects were elicited by peptides spanning the known GT1-specific HLA-B*2705-restricted epitope at position 2841–2849 in NS5B [3] (Table 2).

**Table 2:** GT1 peptides that elicit a response in non-GT1 infected subjects.

HLA	Protein	Position	GT1 peptides tested	Corresponding GT3 consensus sequence	Response with GT3 peptide (n)
**GT1 and GT3 infected subject responders**					
A02	NS2	821	VVLV/a/fGLMAL	GV/iLVLFGFF^#^	Response (1)^@^; *no response* (4)*
A02		822	VLV/fGLMALTL	V/iL/aVLFGFFTL	No response*(2)
A01		836	KVYISWCLW	KHWIGRLIW	Not tested
A02		935	QMAMIKLGAL	QMIILSVGRW^#^	Not tested
A02	NS3	1406	KLVALGLNAV^^^	KLRGMGLNAV^#^	No response*(1)
A01		1436	ATDALMTGY^	ATDALMTGY/f^~^	Yes*
A02	NS4B	1868	IMSGEVPSM^^^	IMGGELPTT/a^#^	Response (1) and no response (1)
B40		1871	GEVPSTEDL^^^	GELPTTEDL	Not tested
B07		1873	VPSMEDLVNL	LPTTEDLVNL	Not tested
A02	NS5A	2252	ILDSFDPLV^^^	ILDSFEPLR^#^	No response*(1)
A24		2280	KFPLAMPVW^^^	KYPPALPIW	*No response (1)*
A11		2281	FT/aPALPIWAR	YPPALPIWAR^#^	No response*(2)
A02		2334	VLTESSVSTA	QLDGSNVSAA^#^	No response* (1)
A02		2338	ST/sVSTALAEL	SNVSAALAAL^#^	No response*(1)
A11	NS5B	2748	GVQEDAASLR	GVDEDRTALR^#^	Not tested
B27		2841	A/vRMIL/mM/lTHF^^^	VRMVMMTHF	*No response (1)*
A01		2858	QLEQALDCEIY	ILDRPLDFEMY	Not tested
A02		2878	DLPP/lIIQRL	DLPAIIERL	Not tested
B44		2939	AICGKYLFNW	KICGLYLFNW	No response*(3)
**GT3 infected subject only responders**					
C04	NS2	848	YFLTRVEAQL	YTICRCES/aAL	No response*(1)
B37		870	RDAVILLM	RDGVILLT	Not tested
B44	NS3	1201	LETTMRSPVF	LSTQARSPSF^#^	Not tested
A11		1265	GAYMSKAH/yGI/v/a^^^	GSFMSRAYGT	No response*(1)
B27		1499	YRFVAPGER	YRYVAPGER^#^	Not tested
B27		1577	KQSGENFPYL	KQQGLNFSYL	Not tested
A11		1636	TLTHPVTK^^^	CLTHPVTK^#^	Not tested
B27	NS5A	2204	SQLSAPSLK	SQLSAPSLK^~^	Yes*
B27	NS5B	2855	ARDQLEQAL	SQEILDRPL^#^	Not tested
B27		2884	QRLHGLSAF	ERLHGLSAF^#^	Not tested
B55		2898	SPGEINRVAA^^^	SPVELNRVAG^#^	Not tested
B44		2924	ARSVRAKLL	ARA/sVRAKLI	No response*(1)
B27		2936	GRAAICGRY^^^	GKAKICGLY^#^	Not tested

^^^known epitope;

^~^consensus sequence the same for GT1 and GT3;

^#^binding prediction from IEDB reduced in GT3 sequence; variants tested separated by dash and indicated by lowercase.

*indicates at least one chronic GT3-infected subject included.

^@^lower response elicited by GT3 peptide than for the GT1 peptide (715 versus 30 SFU/10^6^ PBMCs). Note that where available for the GT3-infected subjects, the autologous sequence matched the testing peptide.

**Fig 4:**
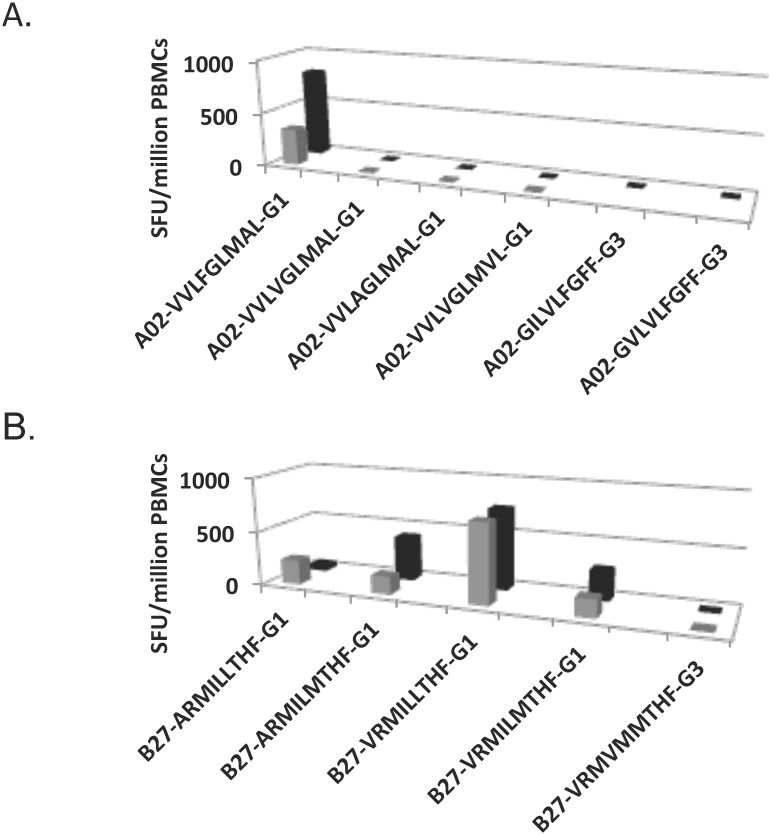
Examples of likely GT-specific responses using ELISpot analysis. The two panels (A) and (B) represent GT1-epitopes that elicited responses from GT3-infected subjects. Peptides representing GT1 peptides tested in the original ELISpot screen and in a subsequent ELISpot with the alternative GT3 peptide(s). Original screen result is shown on the front row (grey) and subsequent ELISpot assay on the second row (black; mean SFU/10^6^ PBMCs from duplicates). Each panel represents data from a single subject.

In order to determine if these GT1 peptides were likely to reflect GT-specific responses, we tested using ELISpot analysis, where possible, the alternative genotype 3 peptide sequences (often corresponding to the autologous virus) in GT 3 chronic-infected subjects that exhibited a response in the initial screen (Table 2). Furthermore, we also tested, where possible, chronic GT1 infected individuals and resolvers with these alternative genotype 3 peptides where a response for a GT1 peptide was detected in the initial screen (Table 2 and Fig 4). These data suggest limited cross-reactivity between these peptides and support our findings that these epitopes are likely to represent GT-specific epitopes and accordingly the responses in the GT3-infected subjects likely represents clearance of a prior exposure.

### Individuals at high-risk of multiple HCV exposure no longer show serological evidence of multiple GT exposure

Evidence of multiple HCV exposures was examined using a HCV serological assay in a subset of 37 chronic HCV-infected subjects and 33 subjects with resolved HCV infection. Of the chronic HCV-infected subjects 25 were GT1-infected and 12 were GT3-infected. Serological testing confirmed 22/25 GT1 infections and 6/12 GT3 infections. There was no evidence of antibodies against alternative GTs in any of the chronic HCV-infected subjects. Of the subjects that underwent HCV serology testing, 10 of the GT3-infected subjects responded to GT1 peptides.

For subjects with resolved HCV infection but with known infecting GT, serological testing confirmed GT1 infection in 6/7, GT2 infection in 2/2 and GT3 infection in 2/5 subjects. For those with unknown infecting GT, the GT could not be determined in 13/20 subjects as the serotyping assay was negative, most likely due to the loss of anti-HCV antibody responses over time. One subject with resolved infection had evidence of dual infection with GT1 and GT2 by serotyping and another subject had been typed as GT3-infected by PCR but was positive for anti-GT1 antibodies by serotyping. Thus, except for two subjects, evidence for dual infection or cleared past infection could not be found by serological analysis, which is consistent with the observation that T-cell responses outlast antibody responses by decades [28, 29].

## Discussion

Genetic studies that identify sites in the viral genome under HLA-restricted host immune pressure (“HLA footprints”) offer an effective way to analyse T-cell responses to viruses. This approach takes viral polymorphisms and population diversity in HLA genes into account and has been applied to HIV vaccine design [7]. Although some studies, including our own, have used a reverse genomics approach to identify a small number of epitopes [24, 30], the approach has not been used to study HCV-specific T-cell responses in a systematic manner. This study provides evidence that HLA-associated viral polymorphisms in the HCV genome can act as markers of in vivo CD8^+^ T-cell selection pressure and can be used to identify novel CD8^+^ T-cell targets and adaptation, and assess GT-specific immunity. As the genetic-guided approach is most helpful in areas of the viral genome that show evidence of viral variation due to lower functional constraint of the target area, this study focussed on the identification of novel epitopes within non-structural genes NS2-NS5B and excluded conserved areas such as the Core/P7 genes that have been extensively mapped for T-cell targets and HCV envelope genes in which sequence variations are driven by antibody rather than T-cell-derived selection pressure. Furthermore, it is likely that the approach used here identified GT-specific epitopes and was used to study GT-specific CD8^+^ T cell responses in subjects with high risk of multiple GT exposure.

As the approach used here identified HLA Class I-restricted T-cell epitopes for HLA-A, -B and -C we were able to compare the response rate of epitopes based on their HLA Class I locus-restriction. These HLA class I loci are closely related and have overlapping functions with respect to T-cell antigen presentation and NK-cell recognition but, aside from peptide binding specificity, subtle differences in these interactions are likely to exist. Furthermore, viruses can affect the expression of the different HLA class loci on the cell surface [31] and the level of expression of these molecules can affect infection outcome [32] suggesting that differences in the influence of the different HLA loci on infection outcome is likely to be reflected in measures of immune pressure on the virus. In HIV, HLA-B-restricted immune responses appear to be the dominant drivers of anti-HIV immunity [10]. Similarly, a study of a single source outbreak [24] identified HLA-B alleles to have a dominant effect on HLA-driven HCV evolution. In this study there were more HLA-B-associated HCV polymorphisms than HLA-A-associated changes, however HLA-A-restricted CD8^+^ T-cell responses were more readily detectable compared with HLA-B-restricted T-cell epitopes. One possible explanation for this observation is that due to the higher heterozygosity value of the HLA-B locus than for the HLA-A locus, lesser numbers of subjects were tested using peptides for any given HLA-B allele relative to HLA-A alleles. For example, most HLA-A*01 and -A*02 predicted T-cell epitopes elicited responses but for these peptides 40 and 63 subjects respectively were tested compared to HLA-B*57 predicted T-cell targets where only 14 subjects were tested with the corresponding peptides. Whilst the probability of observing at least one positive response to a particular epitope is dependent on the numbers tested, the comparisons of the relative response rate of the epitope-specific CD8^+^ T-cell responses accommodate the unequal sample sizes. Unfortunately, due to insufficient numbers of spontaneous resolvers we could not compare HLA locus-specific T-cell responses between individuals with different infection outcome in order to assess the quality of the different HLA locus-restricted responses. Although there is data to suggest HCV affects HLA class I expression [33] the specificity of this host-viral interaction is unknown and accordingly the difference in the bias observed in the genetic leads based on viral adaptation and HLA-loci specific T-cell responses warrants further characterisation.

Results from this study suggested that NS3 and, to a lesser extent, NS5B, genes that code for the viral protease and polymerase, respectively, are most targeted of the non-structural HCV proteins for HLA Class I-restricted CD8^+^ T-cells. T-cell epitopes that lay within these proteins will be of particular importance for HCV vaccine design as research into HIV has shown that T-cell epitopes within proteins with enzymatic functions tolerate limited viral sequence evolution and sequence changes can coincide with reduced viral fitness [34–36]. In the case of HCV, several studies have reported the appearance of “escape” mutations in T-cell epitopes in NS3 and NS5B that were selected by strong CD8^+^ T-cell responses and led to variants with reduced viral replication capacity [3, 36–38]. Although these viral variants might be able to evade one particular CD8^+^ T-cell response, a reduction in virus levels could facilitate immune control initiated by remaining ‘intact’ CD8^+^ T-cell responses against other areas of the genome.

Subjects with chronic HCV infection commonly show evidence for viral escape and responses that are directed against variant/adapted peptides rather than the non-adapted form. Similar findings have been described for HIV [39] and this has since influenced the selection of peptides for vaccine design. This also highlights the fact that HCV can escape natural CD8^+^ T-cell responses and therefore vaccine-induced responses. Accordingly, specific considerations for vaccine design may include incorporating T-cell epitopes, which are not subject to early escape such as ‘subdominant’ epitopes, inclusion of conserved epitopes that are less subject to adaptation and specific exclusion of T-cell epitopes, which appear to have adapted at the population level and are contained within the most common or consensus strains or elicit ineffective responses against the adapted form.

This study also showed that GT1 CD8^+^ T-cell responses were evident in ~38% of chronic non-GT1-infected subjects that have likely encountered previous HCV strains in the past, implying a previously cleared GT1 infection in these subjects. This is further supported by the number of novel GT1 T-cell epitopes identified by including non-GT1 infected subjects in the screening of which several were restricted by the ‘protective’ HLA-B27 allele. The limited cross-reactivity highlighted by ELISpot assays incorporating the alternative GT3 peptides for these GT1 epitopes in subjects with these responses suggests these are GT-specific epitopes. This is further supported by the significant sequence variation between GT1 and GT3 and previous studies comparing immune pressure for the two GTs [4, 9], suggesting these ‘effective’ immune responses are unlikely to be cross-GT specific responses. This finding could also provide an explanation why many previous studies of cellular responses in HCV-infected individuals that did not account for the GT of infection were able to detect T-cell responses using a GT1-based reference sequence, even in subjects with GT3 infection. It is important to note that the peptide panel used in this study was based on viral sequences from the chronic HCV-infected subjects in this study as well as from other subjects in previous studies [8, 9] and should therefore account for the diversity of strains in the autologous viruses of subjects in this study.

The detected HCV GT-specific T-cell responses outlast GT-specific antibody responses, which is consistent with previous studies [28, 29] and therefore CD8^+^ T-cell responses can be used to detect historic infections. The observation of ‘effective’ yet non-cross reactive T-cell responses is not unique to the CD8^+^ T-cell subsets but has also been previously reported in the context of CD4^+^ T-cell responses [40, 41].

We did not make a direct comparison of the breadth and magnitude of CD8^+^ T-cell responses between spontaneous resolvers versus subjects with chronic infection due to the limited number of spontaneous resolvers overall and the likely prior history of exposure in the chronic HCV-infected subjects. Furthermore, as the peptide panel set used in this study does not include all HCV proteins, we could not exclude prior exposure in those subjects with chronic infection that did not respond to peptides from an alternate GT strain.

Finally, this study demonstrates that a reverse-genomics approach, based on the identification of viral adaptation to host’s T-cell responses, can identify T-cell targets and allows effective testing of the anti-viral immune response to circulating viral variants to which the individual was likely exposed in their infection history. The observations described in this study provide an important overview of anti-HCV immunity and viral adaptation necessary for rational immunogen design of a preventative HCV vaccine in the context of multiple GT exposure and to examine the signature of an effective T-cell response against the virus.

## Supporting Information

S1 Table List of peptides tested in study. (DOCX)
