# Supplementary material for: Anti-Hepatitis C Virus T-Cell Immunity in the Context of Multiple Exposures to the Virus
Source: PLoS One. 2015 Jun 24;10(6):e0130420. doi: 10.1371/journal.pone.0130420 (PMC4480353; doi:10.1371/journal.pone.0130420)
Supplement: S1 Table — (DOCX) [file pone.0130420.s001.docx]

**Supplementary Table 1. List of peptides tested in study**

**A. Peptides for predicted epitopes that elicited a response**

| **Protein** | **Start position** | **HLA allele** | **Predicted epitope (and variants tested)** |
| --- | --- | --- | --- |
| **NS2** | **821; 822** | **A*0201** | **VVLV/a/fGLMA/vL; VLV/a/fGLMA/vLTL^** |
|  | **836** | **A*01** | **KR/vYISWCLW** |
|  | **837** | **A*2402** | **RYISWCL/fWWL** |
|  | **848** | **C*0401** | **YFLTRA/t/vEAQ/lL** |
|  | **858** | **A*1101** | **QVWVPPLLAR** |
|  | **870** | **B*3701** | **RDAVILLM** |
|  | **875** | **A*0201** | **LLMCV/aVHPAL** |
|  | **900** | **A*0301** | **WI/vLQASLLK** |
|  | **927** | **C*0701** | **KMAGGHYVQM** |
|  | **935** | **A*0201** | **QMAI/mIKLGAL** |
|  | **940** | **B*1501** | **KL/vGALTGTY** |
|  | **957; 959** | **B*08** | **RDWAHNS/gL; WAHNG/sLRDL** |
| **NS3** | **1073** | **A*0201** | **CI/vNGVCWTV** |
|  | **1111** | **B*0701** | **WPA/sPQGA/sRSL** |
|  | **1201** | **B*4402** | **LETTMRSPVF** |
|  | **1265** | **A*1101** | **GAYMSKAH/yGI/v/a^** |
|  | **1395** | **B*0801** | **HSK/rK/rKCDEL** |
|  | **1402** | **B*0801** | **ELAAKLVAL/m** |
|  | **1436** | **A*0101** | **ATDALMTGY/f^** |
|  | **1492; 1499** | **B*2705** | **GRGKPGIYR; YRF/yVAPGER (predicted only)** |
|  | **1577** | **B*2705** | **KQSGENF/lPYL** |
|  | **1636** | **A*1101** | **TLTHPVTK** |
| **NS4B** | **1725** | **B*3701** | **AEQFKQKAL** |
|  | **1868** | **A*0201** | **IMSGEVPST/m/a** |
|  | **1871** | **B*4001** | **GEV/tPST/m/aEDL** |
|  | **1873** | **B*0702** | **VPST/m/aEDLVNL** |
|  | **1956** | **C*0304** | **LLRRLHQWI** |
| **NS5A** | **2091** | **B*4402** | **VEIRR/qVGDF** |
|  | **2131** | **B*2705** | **HRY/fAPPCKP; HRY/fAPPCKPL** |
|  | **2143** | **B*4001** | **D/eEVSFRVGL^** |
|  | **2164** | **B*3701** | **PEPDVA/tVL/v** |
|  | **2183** | **B*3701** | **AEA/tAGRRL** |
|  | **2192** | **C*0201** | **RGSPPSLA** |
|  | **2204** | **B*2705** | **SQLSAPSLK** |
|  | **2224** | **B*4001** | **A/vELIEANLL** |
|  | **2260** | **B*5101** | **V/rAEEDEREI** |
|  | **2281** | **A*1101** | **FT/aPALPIWAR** |
|  | **2334; 2338** | **A*0201** | **VLTEST/sVSTA; ST/sVSTALAEL** |
|  | **2350** | **C*0304** | **KSFGSSSTSGI/v** |
| **NS5B** | **2459** | **A*1101** | **STTSRSAC/sQR** |
|  | **2748** | **A*1101** | **GVQEDAASLR** |
|  | **2841** | **B*2705** | **A/vRMIL/mM/lTHF** |
|  | **2844** | **A*0201** | **ILMTHFFSV** |
|  | **2855** | **B*2705** | **ARDQLEQAL** |
|  | **2858** | **A*0101** | **QLEQALDCEIY^** |
|  | **2878** | **A*0201** | **DLPP/lIIQRL^** |
|  | **2884** | **B*2705** | **QRLHGLSAF** |
|  | **2923** | **B*0801** | **RARSVRAK/rLL^** |
|  | **2924** | **B*4402** | **ARSVRAK/rLL** |
|  | **2936** | **B*2705** | **GRAAICGK/rY** |
|  | **2939** | **B*4402** | **AICGR/kYLFNW** |

**B. Peptides for predicted epitopes that did not elicit a response**

| **Protein** | **Start position** | **HLA allele** | **Predicted epitope (and variants tested)** |
| --- | --- | --- | --- |
| **NS2** | **814** | **B*5101** | **VAASCGGV/aV** |
|  | **827** | **C*1502** | **MALTLSPHY** |
|  | **852** | **B*5101** | **RAEAQLH/qVW** |
|  | **853** | **B*44** | **AEAQL/qHVWV** |
|  | **926** | **C*0701** | **RKMAGGHYVQM** |
|  | **957** | **B*3701** | **R/kD/nWAHNG/sL** |
|  | **957; 959** | **B*08** | **RDWAHNS/gL; WAHNG/sLRDL** |
| **NS3** | **1383** | **B*5101** | **EA/vIKGGRHLI** |
|  | **1400; 1402** | **B*3701** | **CDELAA/tKL; ELAAKLVAL/m** |
|  | **1492** | **B*2705** | **GRGKPGIYR** |
|  | **1493** | **B*5701** | **RGKP/rGIYRF** |
|  | **1580** | **B*37** | **GENF/lPYLVAY** |
|  | **1620** | **B*13** | **GPTPLLYRL** |
|  | **1635** | **A*68** | **V/iTLTHPVTK** |
| **NS4B** | **1719** | **B*1501** | **EQGMM/aLAEQF^** |
|  | **1725** | **B*3701** | **AEQFKQKAL** |
|  | **1742; 1749** | **C*0401** | **QAEVIT/aPAV; AVQTNWQKL** |
|  | **1799** | **B*0702** | **SPLTTSQTL** |
|  | **1876** | **B*4001** | **T/aEDLVNLL** |
| **NS5A** | **2143** | **B*4001** | **D/eEVSFRVGL^** |
|  | **2192** | **C*0201** | **RGSPPSLA** |
|  | **2421** | **C*0702** | **DTEDVVCSMSY** |
| **NS5B** | **2466** | **B*1501** | **CQ/lRQKKVTF** |
|  | **2504** | **C*0602** | **SVEEACKLT** |
|  | **2510** | **A*31** | **S/dLTPPHSARSK** |
|  | **2538** | **B*08** | **HINSVWKDL** |
|  | **2552** | **A*6801** | **TPIDTTIMAK** |
|  | **2620** | **B*5701** | **RVEFLVQAW** |
|  | **2629** | **B*5701** | **KSKKA/tPMGF** |
|  | **2819; 2821** | **A*25** | **TAK/rHTPVNSW; R/kHTPVNSWLG** |
|  | **2836** | **B*5101** | **APTLWA/vRMI^** |
|  | **2836** | **B*0702** | **APTLWARMIL/m** |

**C. Additional published HCV T-cell epitopes**

| **Protein** | **Start position** | **HLA allele** | **Published epitope (including variants)** |
| --- | --- | --- | --- |
| **NS2** | **827** | **A*29** | **MALTLSPY** |
|  | **910** | **C*03** | **PYFVRAQGLI** |
| **NS3** | **1031** | **A*24** | **AYSQQTRGL** |
|  | **1175** | **A*68** | **HAVGLFRAA** |
|  | **1243** | **C*03** | **AYAAQGYKVL** |
|  | **1359** | **B*35** | **HPNIEEVAL** |
|  | **1391** | **A*03** | **LIFCHSKKK** |
|  | **1406** | **A*02** | **KLVALGINAV** |
| **NS5A** | **2017** | **A*03** | **GVWR/qGD/eGIMH/q** |
|  | **2225** | **A*25** | **DLI/vEANLLW** |
|  | **2252** | **A*02** | **ILDSFDPLV** |
|  | **2266** | **B*40** | **REISVP/aAEIL** |
|  | **2280** | **A*24** | **KFPP/l/sAM/lPI/vW** |
| **NS5B** | **2727** | **A*02** | **GLR/qDCT/iMLV** |
|  | **2794** | **B*38** | **HDGAGKRVYYL** |
|  | **2898** | **B*55** | **SPGEINRVAA** |

^^^ Indicates viral adaptation likely to be consensus sequence

Grey indicates site associated with HLA allele within or flanking known T-cell epitope with same HLA restriction.
